# Study of equivalent circuit of GaN based laser chip and packaged laser

**DOI:** 10.1038/s41598-024-62078-z

**Published:** 2024-05-18

**Authors:** Junfei Wang, Junhui Hu, Chaowen Guan, Yuqi Hou, Leihao Sun, Songke Fang, Jianyang Shi, Ziwei Li, Junwen Zhang, Nan Chi, Chao Shen

**Affiliations:** 1 Key Laboratory for Information Science of Electromagnetic Waves (MoE), School of Information Science and Technology, Fudan University, Shanghai, 200438 China; 2 ZGC Institute of Ubiquitous-X Innovation and Applications, Beijing, 100876 China

**Keywords:** Lasers, LEDs and light sources, Optical materials and structures

## Abstract

High-speed GaN-based lasers play a pivotal role in visible light communication (VLC) systems; however, the causes of the limited modulation response of our fabricated laser diode (LD) are not fully understood. Accordingly, we constructed an equivalent circuit model for both the LD and its packaging. This model enabled us to analyze the series resistance and parallel capacitance of the LD at different injection currents. Experiments and simulations were performed to investigate the intrinsic responses of the LD. The series resistance and parallel capacitance are responsible for S_21_ roll-off at low frequencies. Determination of the packaging design parameters on the modulation response of a transistor outline (TO)-can packaged LD was investigated which is important to achieve the impedance match in the future. The value of each discrete component was determined by fitting the scattering parameters of the equivalent circuit model to the packaged LD. Reducing the series resistance and parallel capacitance improved the modulation response. Our study firstly illustrates the design and manufacture of violet-blue-green laser transmitters with a large modulation bandwidth for ultra-high-speed VLC from the point of the impedance influence.

## Introduction

In recent years, visible-light communication (VLC) has emerged as a prominent technology because of its compelling advantages, such as robust security, cost-effectiveness, immunity to electromagnetic interference, and the freedom of license-free operations^1–4^. VLC is an optical wireless communication technology that uses visible light as a signal source to transmit data^5,6^. Laser diodes (LD) and light-emitting diodes (LED) are widely used optical sources for VLC systems^7–11^. Compared with LEDs, LDs feature a narrow emission spectrum, better output directivity, and higher modulation bandwidth^12–14^. Compared with the near infrared (NIR) laser diode which has already demonstrated the modulation bandwidth exceeding 10 GHz^15^, the modulation bandwidth of visible lasers, such as blue and green LDs based on InGaN/GaN quantum wells (QWs), however, is limited to ~ GHz. which cannot satisfy the escalating data rate demands in VLC^1,9^. The reason for the restricted bandwidth modulation in gallium nitride (GaN) LDs remains unclear. For the determining the primary factors that impede the modulation characteristics of LDs is imperative for high-speed VLC technology^12,16^. Previous studies segmented the entire circuit into three core components: packaging parasitics, chip parasitics, and intrinsic LD chips^17–19^. The influence of the package and chip parasitics on LDs remains unknown. In particular, there is a lack of analysis of the poor modulation response of GaN-based LD. Establishing an equivalent circuit model of the packaged LD and chips helps to extract the intrinsic modulation response of the LD and the influence of parasitics^19–21^. Effectively harnessing the intrinsic LD response will pave the way for optimizing LDs to enhance their modulation characteristics considerably. Here, we discuss the origin of chip and package parasitics and methods to determine their values.

The resistance caused by contacts between GaN and the electrode, particularly in the p-GaN contact and p-cladding layer, along with the capacitance from the multiple quantum wells (MQWs) structure, are significant factors that cannot be overlooked^22–27^. Specifically, chip parasitics, which include both series resistance and parasitic capacitance, vary under different bias conditions^26^. The varying impedance of the chip parasitics also affects the overall modulation response of the LD. Therefore, a method to abstract chip parasitics as they change with the injection current and to mitigate their influence needs to be developed to deduce the intrinsic scattering parameters accurately. The impedance of the entire network can be determined by using a vector network analyzer (VNA) to measure the scattering parameters. Calibrating the probe to set the reference plane at the probe tip is essential during the scattering parameter test^28^. Once an equivalent circuit model of the chips is established and the component values are ascertained, the influence of parasitics can be effectively determined.

In addition to chip parasitics, package parasitics are limiting factors related to the modulation response. Most GaN-based LDs are encapsulated in a transistor outline (TO)-can, which is typically employed as a standard packaging method. The electrically driven signal is transmitted through the TO lead and bonding wire, both of which inevitably introduce impedance into the pulse signal relayed to the LD chip^29^. However, the influence of packaging components on the modulation response is yet to be explored. Impedance mismatches lead to a significant return loss, which in turn impedes the transmission of the modulated signal to the chips^30^. Consequently, developing a method to characterize parasitics in packaging has emerged as a pressing necessity. The VNA can be meticulously calibrated to the extremities of the coaxial cable using the short-open-match method. This calibration can be further extended to the end of the TO lead using a de-embedding technique^29^. After establishing an equivalent circuit model for the packaged LD, we can effectively determine the value of the discrete parts within the package network. This crucial step lays the foundation for successful impedance matching in GaN-based LDs.

In this study, we formulated equivalent circuit models for both LD chips and their packaged counterparts. Our study emphasized the construction of a dedicated model for LD chips, aiming to assess the component values associated with chip parasitics meticulously. This aspect is critical for extracting the intrinsic response of LDs. In addition, we propose a specific circuit model for packaged LDs for the accurate determination of the values of the different elements involved in packaging. This dual approach enables a comprehensive analysis, facilitating deeper insight into both the intrinsic and extrinsic factors that affect the performance of LDs. Our experimental investigation involved measuring the scattering parameters for both LD chips and packaged LDs. This was accomplished following the calibration of the VNA and a critical de-embedding process at the test port, which was necessary to ensure the precision of our scattering parameter results. By analyzing the experimental scattering parameter data, we could isolate and determine the individual component values associated with both package and chip parasitics. The deduction of the component values is necessary to determine which component contributes significantly to the deterioration of the modulation response. We derived the intrinsic modulation response of the LD by integrating these lumped elements into our equivalent circuit model. The extraction of the intrinsic modulation response is critical for the design of a high-speed GaN-based violet-blue-green laser. Moreover, our study can be extended to changing the value of chip parasitics and identifying potential areas for optimization through refined processing techniques.

### Model and theoretical analysis

**Figure 1:**
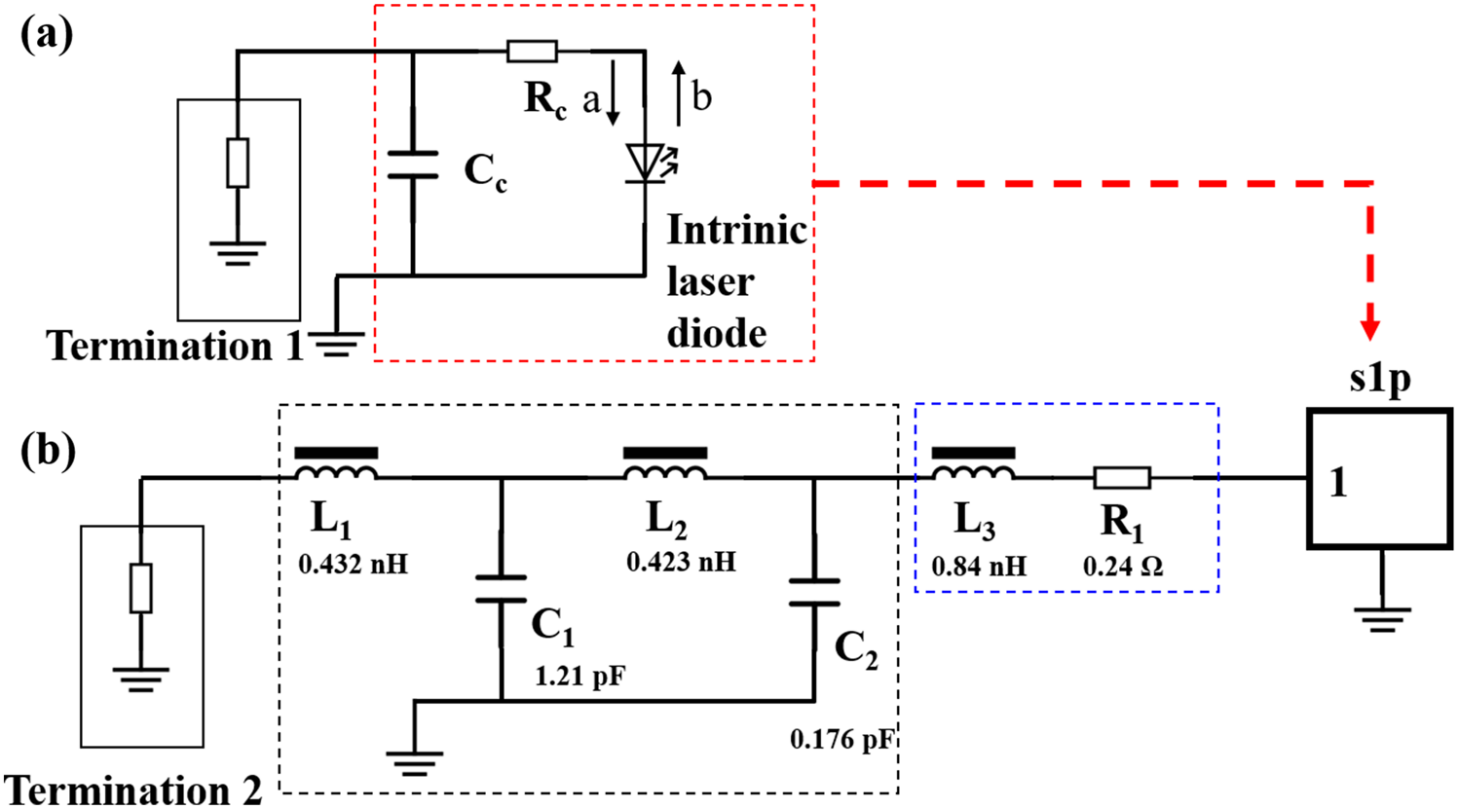
(**a**) Equivalent circuit of the LD chip. R_c_ and C_c_ are the series contact resistance and parallel capacitance, depicted in the red-colored dotted line. Termination 1 refers to the test port of the experimental equipment. The chip network includes an intrinsic LD cascaded with R_c_ and C_c_. (**b**) Equivalent circuit of the packaged LD. L_1_, L_2_ and C_1_, C_2_ are the inductors and capacitance originating from the TO lead depicted in the black-colored dotted line. L3 and R1 are the inductor and resistance originating from the bonding wire, depicted in the blue-colored dotted line. s1p refers to the LD chip. Termination 2 represents the test port of the experimental equipment.

In this study, we determined the intrinsic response of an LD by constructing an equivalent circuit model excluding packaging influences, as illustrated in Fig. 1a. In this model, R_c_ represents the series resistance of the laser chips, primarily arising from the contact resistance between the p-GaN with the metal electrode and the p-GaN layer. C_c_ represents the total parallel capacitance of the device, and is a key factor in determining its operational characteristics. Notably, when a positive bias is applied, carrier diffusion and accumulation in the quantum well become the predominant components of this parallel capacitance. The parasitic components of the model are subject to changes under variations in the bias current. A thorough two-port coaxial port calibration was performed to capture the scattering parameters (S-parameters) of the entire model accurately. This calibration process, which included the extended open short-load method and employed a corresponding calibration kit (MPI-T40), was crucial for advancing the test plane to the probe tips. In this conceptualization, we treat the circuit as an intrinsic LD coupled with its parasitic elements, specifically C_c_ and R_c_, thereby facilitating a more precise understanding of the diode behavior.

As mentioned earlier, the T matrix of the entire model can be expressed as^31^

1 $$\begin{array}{*{20}c} {T^{{\text{D}}} = T^{{\text{P}}} T^{{\text{L}}} = \frac{1}{{S_{21}^{{\text{L}}} S_{21}^{{\text{P}}} }}\left[ {\begin{array}{*{20}l} 1 \hfill & { - S_{22}^{{\text{P}}} } \hfill \\ {S_{11}^{{\text{P}}} } \hfill & {S_{12}^{{\text{P}}} S_{21}^{{\text{P}}} - S_{11}^{{\text{P}}} S_{22}^{{\text{P}}} } \hfill \\ \end{array} } \right]\left[ {\begin{array}{*{20}c} 1 & 0 \\ {S_{11}^{{\text{L}}} } & 0 \\ \end{array} } \right]} \\ \end{array}$$

In the subscripts, ${\text{D}}$ indicates the entire circuit, including the parasitic and intrinsic LDs, ${\text{P}}$ represents the parasitic of LDs, and ${\text{L}}$ denotes the intrinsic LDs. By transforming the above T-matrix into S-parameters, we obtain^31^

2 $$\begin{array}{*{20}c} {S^{{\text{D}}} = \frac{1}{{T_{11}^{{\text{D}}} }}\left[ {\begin{array}{*{20}l} {T_{21}^{{\text{D}}} } \hfill & {T_{12}^{{\text{D}}} T_{21}^{{\text{D}}} - T_{11}^{{\text{D}}} T_{22}^{{\text{D}}} } \hfill \\ 1 \hfill & { - T_{12}^{{\text{D}}} } \hfill \\ \end{array} } \right]} \\ \end{array}$$

Combining Eqs. (1) and (2), the S-parameters of the intrinsic LDs can be expressed as^31^

3a $$\begin{array}{*{20}c} {S_{11}^{{\text{L}}} = \frac{{S_{11}^{{\text{D}}} - S_{11}^{{\text{P}}} }}{{S_{12}^{{\text{P}}} S_{21}^{{\text{P}}} + S_{22}^{{\text{P}}} \left( {S_{11}^{{\text{D}}} - S_{11}^{{\text{P}}} } \right)}}} \\ \end{array}$$

3b $$\begin{array}{*{20}c} {S_{21}^{{\text{L}}} = \frac{{1 - S_{22}^{{\text{P}}} S_{11}^{{\text{L}}} }}{{S_{21}^{{\text{P}}} }}S_{21}^{{\text{D}}} } \\ \end{array}$$

Compared with the series resistance (R_c_), we can regard the intrinsic LD as a short port because of its lower resistance. Hence, the reflection coefficient of the intrinsic LD could be considered as -1. Therefore, Eq. (3a) can be transformed into

4 $$\begin{array}{*{20}c} {S_{11}^{{\text{D}}} = S_{11}^{{\text{P}}} - \frac{{S_{21}^{{\text{P}}} S_{12}^{{\text{P}}} }}{{1 + S_{22}^{{\text{P}}} }}} \\ \end{array}$$

In this equation, $S_{11}^{{\text{D}}}$ can be tested after calibration using a VNA. $S_{11}^{{\text{P}}}$, $S_{12}^{{\text{P}}}$,${ }S_{21}^{{\text{P}}}$, and $S_{22}^{{\text{P}}}$ can be expressed as functions of R_c_ and C_c_. Consequently, the $S_{11}^{{\text{D}}}$ of the entire circuit, along with the frequency, is supposed to change with the parasitics. By fitting the test results of $S_{11}^{{\text{D}}}$ under different injection currents, the parasitic behavior of the model could be obtained. By substituting the S-parameters of the parasitics and $S_{21}^{{\text{D}}}$ into Eq. (3b), the modulation characteristics of the intrinsic LD ($S_{21}^{{\text{L}}}$) can be calculated.

The injection current of the intrinsic LD can be expressed as

5 $$\begin{array}{*{20}c} {i_{A} = \frac{{\left( {a - b} \right)}}{{\sqrt {Z_{r} } }}} \\ \end{array}$$

where $a$ is the amplitude of the incident voltage wave, $b$ is that of the reflected voltage wave, and $Z_{r}$ is the characteristic impedance. Therefore, the relationship between S_21_ and the current modulation coefficient is

6 $$\begin{array}{*{20}c} {\eta_{L} \left( f \right) = \sqrt {Z_{r} } \frac{{S_{21}^{L} }}{{1 - S_{11}^{L} }}} \\ \end{array}$$

7 $$\begin{array}{*{20}c} {\eta_{D} \left( f \right) = \sqrt {Z_{r} } \frac{{S_{21}^{D} }}{{1 - S_{11}^{D} }}} \\ \end{array}$$

Here, $\eta_{L} \left( f \right)$ is the current modulation coefficient of the intrinsic LD, and $\eta_{D} \left( f \right)$ is the current modulation coefficient of the LD chip.

Owing to the negligible resistance of the intrinsic laser, $S_{11}^{{\text{L}}}$ can be regarded as -1. By combining Eqs. (3b), (6), and (7), we obtain

8 $$\begin{array}{*{20}c} {\frac{{\eta_{{\text{D}}} }}{{\eta_{{\text{L}}} }} = \frac{{2S_{21}^{{\text{P}}} }}{{\left( {1 - S_{11}^{{\text{P}}} } \right)\left( {1 + S_{22}^{{\text{P}}} } \right) + S_{12}^{{\text{P}}} S_{21}^{{\text{P}}} }}} \\ \end{array}$$

Therefore, once the parasitics are identified, their influence on the entire network can be evaluated.

In the context of application systems, the characteristic impedance is typically 50 Ω. However, this standard leads to a significant impedance mismatch with the LD, resulting in a severe mode-field mismatch. A crucial step in addressing this challenge is to determine the discrete component impedance within the package network accurately. In our experimental setup, we constructed an equivalent circuit model for the package network, as shown in Fig. 1b. In this model, L_1_ and L_2_ represent the inductors originating from the TO leads, whereas C_1_ and C_2_ are the capacitors associated with these leads. L_3_ and R_1_ denote the inductor and resistor originating from the gold wire used to connect the package to the chip electrode, respectively. The LD is represented as ‘s1p’ in the schematic, which correlates with Fig. 1a, and Termination 2 is identified as the test port for the operational system. Our network model segments the entire circuit into two parts: package parasitics and an LD chip in a cascading arrangement. The S-parameter for s1p was established as described above, and the S-parameter for the entire network could be assessed after the calibration of the VNA. Utilizing the advanced design system (ADS) for network simulation, we observed that modifications to package parasitics, including changes to L_1_, L_2_, C_1_, C_2_, L_3_, and R_1_, significantly influenced the S-parameter of the entire network. By aligning the simulation results of S_11_ with our experimental data, we could determine the exact values of these discrete components. Finally, the S-parameters of the parasitic network were deduced by considering the network as a “π-shaped” configuration.

## Experimental results and discussions

**Figure 2:**
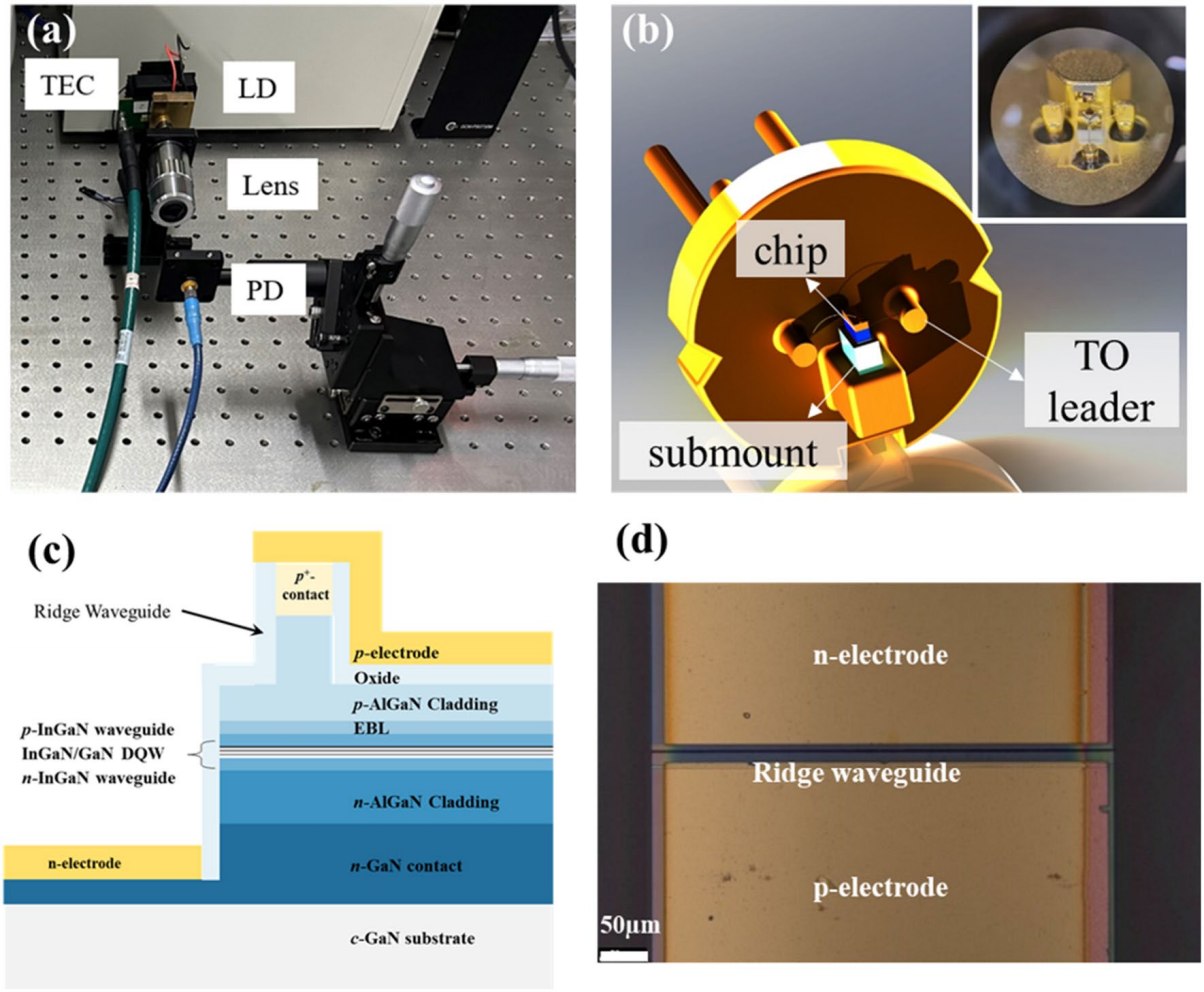
(**a**) Schematic of the S-parameter test system; The packaged LD is mounted on the designed driver with a thermoelectric cooler (TEC) to ensure the stable operation of the laser. (**b**) Schematic of the packaged LD. The laser chip is mounted on the submount through bonding. Insert of (**b**): Optical microscope image of TO-can packaged LD. (**c**) Schematic of the epitaxial layer of the fabricated LD chip. (**d**) Picture of the fabricated chip. The n- and p-electrodes marked in the picture are located on either side of the ridge.

In this study, we established an S-parameter measurement system as illustrated in Fig. 2a. The system was segmented into three primary sections: transmitter, which is an LD; receiver, which is a photodiode (PD); and VNA. Initially, a small sinusoidal signal generated from Port 1 was directed toward the LD. Upon reflection, the signal was recaptured using the same port (Port 1). Within the LD, the electrical signal was merged with the direct current (DC) through the Bias Tee (TCBT-203+) and converted to an optical form. The driving current was transmitted through a gold line connected to the TO leader sent to the chip. This optical signal was then received by the PD, where it was transformed back into an electrical signal, and subsequently transmitted to Port 2. As the − 3 dB modulation bandwidth of the PD was 10 GHz or higher, we could regard the modulation response of the PD as a constant. The modulation response of the entire system effectively mirrored that of the LD. The LD chip illustrated in Fig. 2b was mounted on an aluminum nitride (AlN) submount and connected to the TO leader via a gold wire. The detailed structure of the LD chip, specifically, its epitaxial layer, is shown in Fig. 2c. Figure 2d shows a microscopic image of the LD chip.

**Figure 3:**
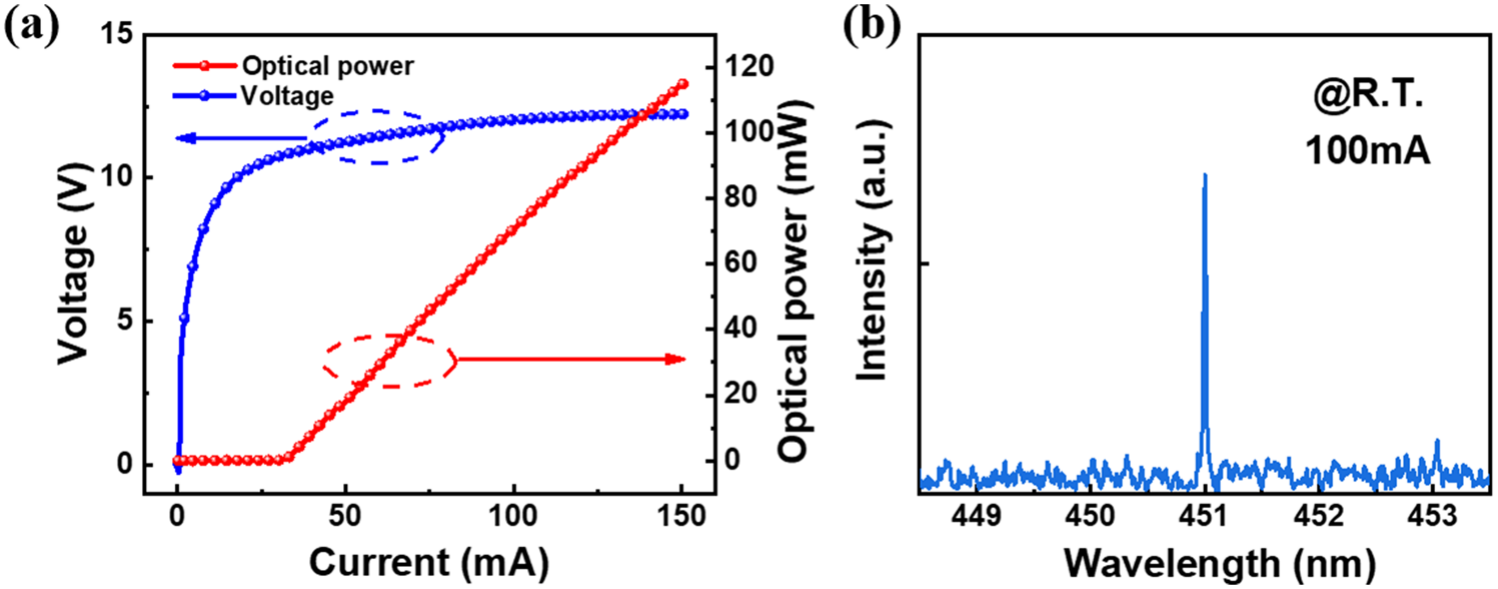
(**a**) Light–current–voltage (L–I–V) characteristics of the laser under the condition of continuous wave (CW) injection. (**b**) Spectra of the laser diode under 100 mA injection current at room temperature.

The light–current–voltage (L–I–V) characteristics of the laser under continuous wave injection current is shown in Fig. 3a. Ridge width of the fabricated LD chip is 1.8 μm and the cavity length of the LD chip is 500 μm. Threshold current density of the LD chip is 3.4 kA/cm^2^ and the slope efficiency is 1.01W/A. Emission spectra of the laser diode under 100 mA injection current is shown in Fig. 3b. Centre wavelength is 451 nm.

**Figure 4:**
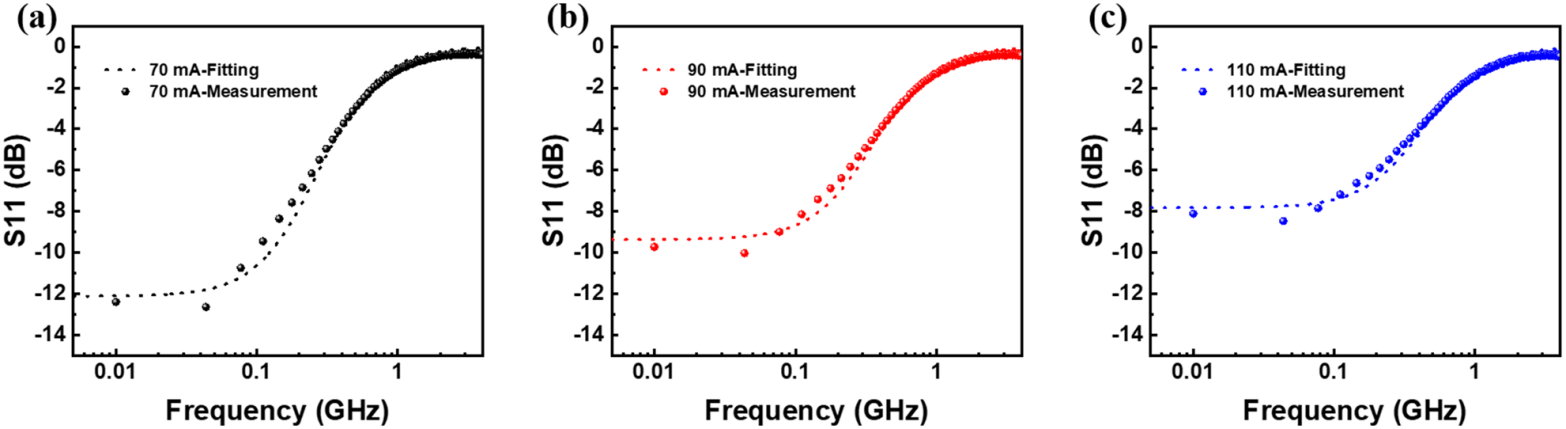
S_11_ parameter varying with the small-signal frequency at the injection currents of 70 mA (**a**), 90 mA (**b**), and 110 mA (**c**). The dots in the figure represent the experimental results, and the dashed lines represent the simulation results.

After calibrating the PNA-L Network Analyzer N5230C for alignment with the probe tips, we precisely measured the return loss (S_11_), as illustrated in Fig. 4. The black, red, and blue traces represent the biased currents of 70 mA (a), 90 mA (b), and 110 mA (c), respectively. We also built a chip circuit using the ADS software. During our simulation, a broad range of values was assigned to the variables R_c_ and C_c_. The specific values for these variables were determined based on the fitting accuracy of S_11_. The dots depicted in the figure represent our experimental results, whereas the dashed lines represent the simulation results. Remarkably, there is excellent consistency between the test data and simulation outcomes, which not only reinforces the accuracy of our model but also substantiates the precise values extracted from our circuit.

**Figure 5:**
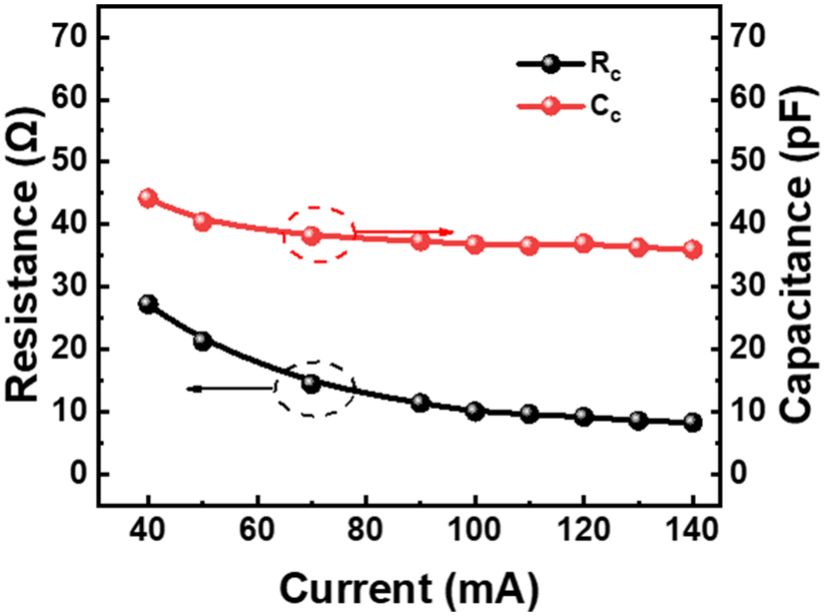
Extracted values of the parasitic resistance (R_c_) and capacitance (C_c_) in the equivalent model shown in Fig. 1a under an injection current ranging from 50 to 140 mA. The red trace represents the variation in the capacitance, and the black trace represents the variation in the resistance.

As illustrated in Fig. 5, there is a noticeable decrease in the resistance with an increase at the injection current above the threshold current. This trend is consistent with the *I*–*V* characteristic traces observed in our experiments. The decrease in series resistance is attributed to the fact that the diffusion velocity of the carriers into the MQWs follows an exponential relationship, which facilitates the efficient recombination of carriers in the active region. The capacitance decreases at the start of the lasering situation, then it keeps constant around the injection current above 70 mA, which is still under investigated.

**Figure 6:**
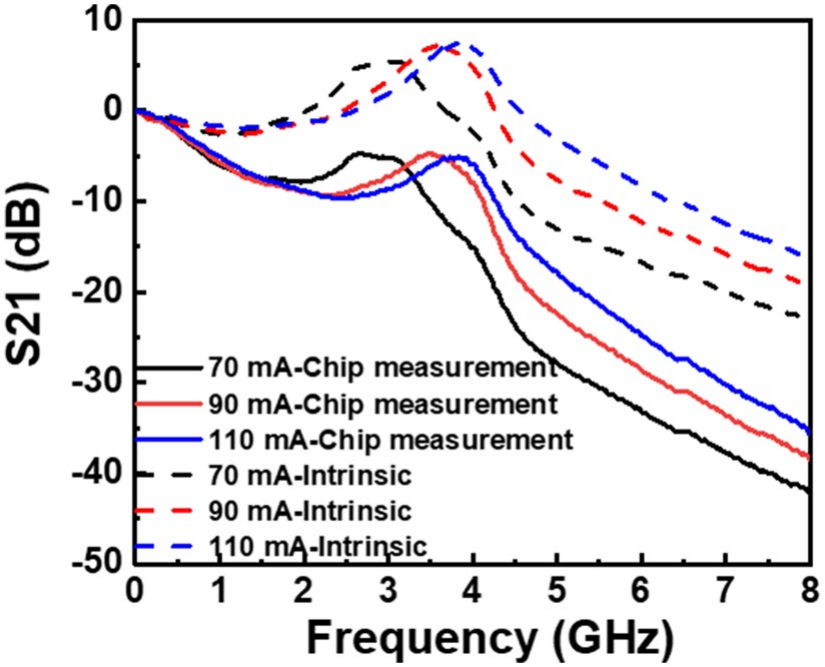
Small-signal modulation response of the LD chip under an injection current ranging from 70 to 110 mA. The dotted lines indicate the corresponding intrinsic modulation response.

Following the extraction of the parasitics of the LD, we obtained the intrinsic small-signal modulation characteristics, as defined in Eq. (3b). The measured modulation properties (S_21_) at different injection currents, along with their respective intrinsic modulation responses, are shown in Fig. 6. A prominent roll-off was observed in the low-frequency domain (below 1 GHz) of the LD chip, largely owing to the influence of the parasitic resistor and capacitance.

**Figure 7:**
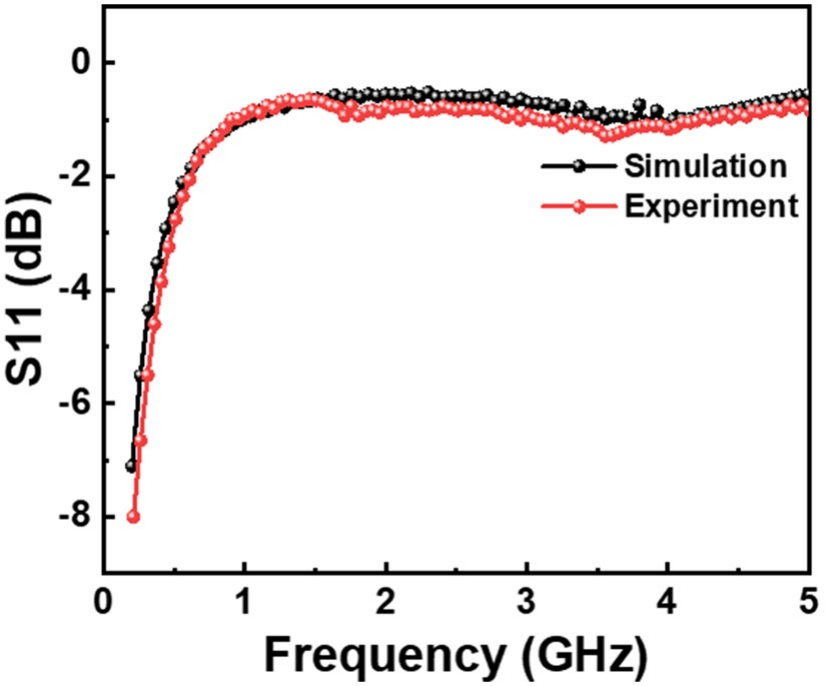
S_11_ parameter of the packaged LD varying with the signal frequency at the injection current of 70 mA. The red curve shows the test result, and the black curve shows the simulation result.

In addition to the parasitics of the LDs, the package parasitics significantly affect the transmission characteristics. Therefore, we established an equivalent circuit for the packaged LD, as illustrated in Fig. 1b. In this circuit, L_1_ and L_2_ refer to the inductors originating from the TO leader, and C_1_ and C_2_ denote the capacitances linked to the TO lead. L_3_ and R_1_ represent the inductor and resistor, respectively, which are attributed to the bonding wire. The s1p in this figure symbolizes the LD chip, and Termination 2 indicates the test port. Integrating the parameters of the LD chip into s1p and varying the package parasitics led to changes in the overall package network. The precise values of the package components are determined by fitting the simulation results to an S_11_ test trace as shown in Fig. 7. The red curve displays the test results, whereas the black curve represents the simulation data. The remarkable consistency between these results confirms the accurate extraction of package parasitic component values. The specific values of these components are listed in Fig. 1b.

**Figure 8:**
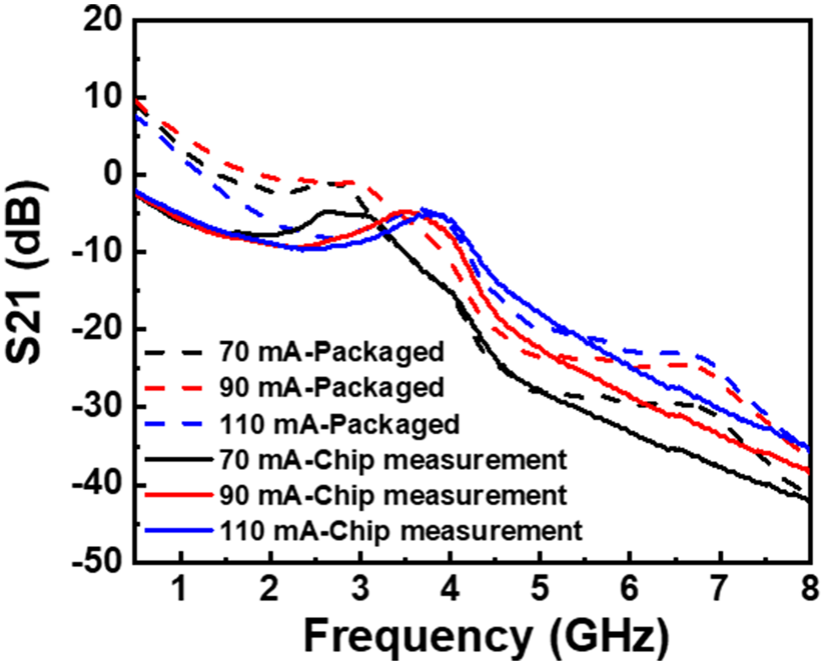
Small-signal modulation response of the LD chip and the packaged LD under different injection currents.

The effect of package parasitics can be observed in the small-signal modulation characteristics shown in Fig. 8. Following the packaging process, a steeper roll-off was observed at relatively low frequency, and there was a noticeable reduction in the resonance frequency.

**Figure 9:**
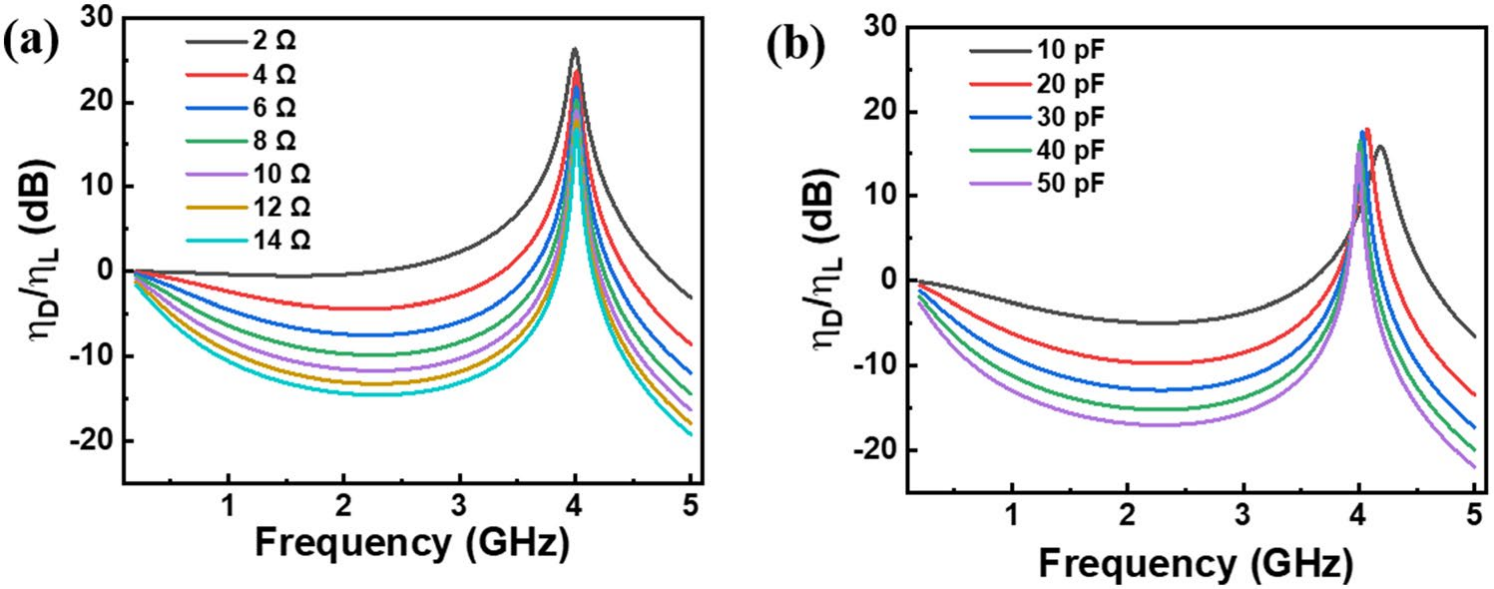
(**a**) Influence of the chip parasitic resistance (varying from 2 to 14 Ω) on the small-signal modulation characteristics. ${\eta }_{{\text{D}}}/{\eta }_{{\text{L}}}$ represents the LD modulation response deviated from the intrinsic modulation response. (**b**) Influence of the chip parasitic capacitance (varying from 10 to 50 pF) on the small-signal modulation characteristics.

Following the determination of package and chip parasitics, their influence on the modulation properties became evident. Although package parasitics are associated with certain standards that affect other results in addition to the modulation characteristics, we specifically investigated the influence of varying chip parasitics on small-signal modulation characteristics. Using Eq. (8), we analyzed the differential effects between ${\eta }_{{\text{D}}}$ and ${\eta }_{{\text{L}}}$. As shown in Fig. 9a, an increase in the series resistance is correlated with a marked decrease in the modulation response. Furthermore, as shown in Fig. 9b, an increase in the parallel capacitance leads to a reduction in both the modulation response and resonance frequency. Thus, we could acknowledge the influence of the series resistance and parallel capacitance on the modulation response of the GaN-based LD. Furthermore, reducing both the series resistance and parallel capacitance could be an effective strategy for improving the modulation response for GaN based LD chip. Here we imposed some possible solutions along with the fabrication of the GaN-based LD. The high specific contact resistance between the metal electrode and p-GaN, coupled with the considerable bulk resistance of p-GaN, are the key hindrances to achieving a low series resistance. To overcome these limitations, it is crucial to increase the p-doping concentration and optimize the p-electrode composition. Such improvements aim to reduce the barrier thickness at the interface, thereby diminishing the series resistance and enhancing the modulation response. In terms of the parallel capacitance, narrowing the ridge width and reducing the cavity length are effective methods for decreasing the active region area, which can reduce the capacitance.

## Conclusions

We presented a comprehensive equivalent circuit model for GaN-based LDs and analyze their frequency responses. The S_11_ parameter of the LD chip reflects its parasitic elements. By calculating the S_11_ of the equivalent circuit of the chip, we can accurately extract the series resistance and parallel capacitance of the chip. This crucial step helps us investigate the intrinsic response of the GaN-based LD. The RC parasitic effect on the modulation response has been investigated, leading to a clear picture on understanding the limited factor of the modulation response in nitride lasers. In addition, by integrating the S_11_ data of the chip into the overall package network and fitting them to the S_11_ data of the entire package network, we can identify the specific component values within the packaged laser network. This study serves as a foundational step toward achieving impedance matching in GaN-based LDs.

## Data Availability

The datasets analyzed in the current study are available from the corresponding author on request.
